# Chromosome-Level Genome Sequence of Aspergillus chevalieri M1, Isolated from Katsuobushi

**DOI:** 10.1128/MRA.00385-21

**Published:** 2021-09-16

**Authors:** Chihiro Kadooka, Kazuki Mori, Kayu Okutsu, Yumiko Yoshizaki, Kazunori Takamine, Kosuke Tashiro, Hisanori Tamaki, Taiki Futagami

**Affiliations:** a Education and Research Centre for Fermentation Studies, Faculty of Agriculture, Kagoshima University, Kagoshima, Japan; b United Graduate School of Agricultural Sciences, Kagoshima University, Kagoshima, Japan; c Cell Innovator Co., Ltd., Fukuoka, Japan; d Laboratory of Molecular Gene Technology, Faculty of Agriculture, Kyushu University, Fukuoka, Japan; Vanderbilt University

## Abstract

In this study, we report the chromosome-level genome sequence of the osmophilic filamentous fungus Aspergillus chevalieri M1, which was isolated from a dried bonito, katsuobushi. This fungus plays a significant role in the fermentation and ripening process. Thus, elucidating the sequence data for this fungus will aid in subsequent genomic research on the fungi involved in katsuobushi production.

## ANNOUNCEMENT

Naturally occurring fungi have been used in the traditional production of dried bonito, katsuobushi, in Japan. The fungi play a significant role in the degradation of proteins and lipids, as well as the formation of flavor during the production of katsuobushi. In a previous study, we analyzed the fungal flora transition during katsuobushi production and isolated Aspergillus chevalieri (1). A. chevalieri isolates were further classified into teleomorphic and anamorphic strains, based on morphological analysis. The teleomorphic strain was dominant throughout the katsuobushi production (1). A. chevalieri was also identified in different katsuobushi samples in Japan (2). In addition, A. chevalieri was isolated from fermented foods such as bagoong, a traditional fermented fish prepared with salted anchovy from Luzon Island in the Philippines (3), and meju, a brick of dried fermented soybeans in Korea (4).

To understand the fungi used for the production of katsuobushi, we sequenced the genome of the teleomorphic A. chevalieri strain M1 (1). Strain M1 was cultivated in M40Y medium (40% [wt/vol] sucrose, 2% [wt/vol] malt extract, 0.5% [wt/vol] yeast extract, and 2% [wt/vol] agar) (DSMZ medium 187). Then, the mycelia retrieved were subjected to DNA extraction using DNAs-ici!-F (Rizo Inc., Tsukuba, Japan) and RNA extraction using RNAiso Plus (TaKaRa Bio Inc., Shiga, Japan) and the SV total RNA isolation system (Promega, Madison, WI). The genomic DNA of strain M1 was sequenced to 178-fold and 179-fold coverage using Oxford Nanopore Technologies (ONT) MinION and Illumina NovaSeq 6000 platforms, respectively, with PCR-free workflows. ONT and Illumina sequencing libraries were prepared using the ligation 1D kit (SQK-LSK109; ONT) and the NEBNext Ultra II DNA library preparation kit (E7645; New England BioLabs [NEB]), respectively. The ONT reads and Illumina reads were used for *de novo* assembly and error correction, respectively. The ONT reads were assembled by Canu v2.0 (5), Flye v2.8-b1674 (6), and Raven v1.5.0 (7). The Flye and Raven assemblies were used to bridge separate contigs generated by Canu. We choose the better assembly metrics based on telomere-to-telomere genome assembly. Consequently, the genome of strain M1 was assembled into 9 contigs, consisting of 8 chromosomes and 1 mitochondrial DNA, thus indicating that we successfully sequenced the nearly complete genome sequences of strain M1. Five chromosomes were generated only by Canu, while chromosomes 3, 7, and 8 were generated by an assembly in which 2 Canu contigs were bridged by Flye and Raven contigs. The final assembly was polished using medaka v1.0.3 (8) with ONT reads, Pilon v1.23 (9) with ONT reads, and Pilon v1.23 (9) with Illumina reads. The genome annotation of the chromosomal contigs and mitochondrial contig obtained was performed based on the Funannotate v1.8.1 pipeline (10) and MFannot v1.1 (11), respectively. Gene prediction was performed by using SNAP v2006-07-28 (12), AUGUSTUS v3.3.3 (13), GlimmerHMM v3.0.4 (14), and GeneMark-ES v4.61_lic (15) via the Funannotate v1.8.1 pipeline (10). For the analysis, RNA-seq reads for strain M1 were obtained with the NovaSeq 6000 system and used for gene prediction. The sequencing library was prepared using the NEBNext Ultra directional RNA library preparation kit for Illumina (E7420; NEB). The Illumina reads were *de novo* assembled by Trinity v2.8.5 (16) and analyzed with the sequence alignment tool HISAT v2.2.0 (17). The proteins were annotated with MEROPS v12.0 (18), UniProt v2020_05 (19), MIBiG v1.4 (20), Pfam v33.1 (21), and dbCAN2 v9.0 (22) (based on the CAZy database v7/30/2020 [23]) using sequence alignment tools such as DIAMOND v2.0.6 (24) and HMMER v3.3.2 (25). Annotation was also performed using InterProScan v5.47-82.0 (26), eggNOG-mapper v1.0.3 (27) (for the EggNOG v4.5 database [28]), antiSMASH v5.1.2 (29), SignalP v4.1 (30), Phobius v1.01 (31), tRNAscan-SE v2.0.7 (32), and Barrnap v0.9 (33). The nearly complete genome of strain M1 includes 29,748,498 bp, with a GC content of 49.2%, and is composed of 10,356 predicted coding sequences and 196 tRNAs. Genome completeness was assessed using Benchmarking Universal Single-Copy Orthologs (BUSCO) v5.1.2 with the ascomycota_odb10 data set (34), which resulted in 97.9% complete and single-copy BUSCOs, 0.4% complete and duplicate-copy BUSCOs, 0.5% fragmented-copy BUSCOs, and 1.2% missing BUSCOs. The details regarding the replicons present are summarized in Table 1. The chromosome-level genome sequence data for strain M1 will allow detailed genomic characterization to determine the possible applications and importance of A. chevalieri in fermentation industries.

**TABLE 1:** Replicons of Aspergillus chevalieri M1

Location^*a*^	GenBank accession no.	Size (Mb)	GC content (%)	No. of coding sequences	No. of rRNAs	No. of tRNAs
Chr. 1	AP024416.1	5.59	49.2	1,944	0	28
Chr. 2	AP024417.1	4.31	49.2	1,471	0	36
Chr. 3	AP024418.1	4.1	49.3	1,423	0	21
Chr. 4	AP024419.1	3.79	49.4	1,366	0	18
Chr. 5	AP024420.1	3.72	49.4	1,312	0	24
Chr. 6	AP024421.1	2.96	49.2	1,043	0	11
Chr. 7	AP024422.1	2.8	49.3	943	27 (82)^*b*^	11
Chr. 8	AP024423.1	2.43	49.1	839	0	20
MT	AP024424.1	0.05	28.1	15	1	27

a Chr., chromosome; MT, mitochondria.

b The number of rRNA genes is not clear due to their highly repetitive structure. The number in parentheses indicates the estimated copy number based on the median per-base coverage.

### Data availability.

The nucleotide sequences of A. chevalieri M1 chromosomes and mitochondria have been deposited in DDBJ/ENA/GenBank under accession numbers AP024416 to AP024424. Raw sequence reads have been deposited in the Sequence Read Archive (SRA) under accession numbers DRX256206, DRX251720, and DRX251721.
